# The Influence of Organizational Climate on the Enrollment of People of Color Into Clinical Trials

**DOI:** 10.1093/geroni/igaa057.1067

**Published:** 2020-12-16

**Authors:** Marcela Blinka, Karlynn BrintzenhofeSzoc, James Zabora

**Affiliations:** 1 Johns Hopkins University, School of Medicine, Baltimore, Maryland, United States; 2 University of Cincinnati, Cincinnati, Ohio, United States; 3 Sidney Kimmel Comprehensive Cancer Center at JH, Baltimore, Maryland, United States

## Abstract

A significant need exists to improve enrollment of people-of-color (POC) in randomized clinical trials as the gold standard for evaluating new therapies so that health disparities can be reduced. Improving representation in clinical trials is expected to improve the applicability of clinical findings more broadly, especially for minority subgroups. This cross-sectional study assessed the perception of organizational climate (OC) among staff in five cancer research groups (CRGs) of a mid-Atlantic NCI-designated comprehensive cancer center. The Organizational Climate Measure (OCM), a measure of four OC models of the Competing Values Framework, was used. Enrollment data were obtained from the 2016 Cancer Research Management System data and 2017 enrollment rates. A statistically significant difference in mean scores was found in the Pressure to Produce Scale (F = 4.21, p < .05). One CRG’s staff reported lower pressure to meet targets than three other CRGs (p < .05) and trending towards significance for the fourth CRG (p < .08). This suggests that organizational priorities considering all patients as potential clinical trial participants increases accrual. A statistically significant negative relationship between the Outward Focus sub-scale and POC enrollment (r = -.228, p < .05) was also found, suggesting that an external organizational focus, alone, is insufficient to increase POC accrual. Implications for future research include 1) does the organizational priority of decreasing the pressure to produce influence accrual of POC? and 2) does an OC that is flexible and externally focused lead to a more effective means of accruing POC as clinical trial participants?

